# A Study of Hand Back Skin Texture Patterns for Personal Identification and Gender Classification

**DOI:** 10.3390/s120708691

**Published:** 2012-06-26

**Authors:** Jin Xie, Lei Zhang, Jane You, David Zhang, Xiaofeng Qu

**Affiliations:** Biometrics Research Center, Department of Computing, The Hong Kong Polytechnic University, Kowloon, Hong Kong; E-Mails: csjxie@comp.polyu.edu.hk (J.X.); csjyou@comp.polyu.edu.hk (J.Y.); csdzhang@comp.polyu.edu.hk (D.Z.); csxfqu@comp.polyu.edu.hk (X.Q.)

**Keywords:** biometrics, hand back skin texture, texton learning, sparse representation

## Abstract

Human hand back skin texture (HBST) is often consistent for a person and distinctive from person to person. In this paper, we study the HBST pattern recognition problem with applications to personal identification and gender classification. A specially designed system is developed to capture HBST images, and an HBST image database was established, which consists of 1,920 images from 80 persons (160 hands). An efficient texton learning based method is then presented to classify the HBST patterns. First, textons are learned in the space of filter bank responses from a set of training images using the *l*_1_ -minimization based sparse representation (SR) technique. Then, under the SR framework, we represent the feature vector at each pixel over the learned dictionary to construct a representation coefficient histogram. Finally, the coefficient histogram is used as skin texture feature for classification. Experiments on personal identification and gender classification are performed by using the established HBST database. The results show that HBST can be used to assist human identification and gender classification.

## 1. Introduction

Skin, as the outermost part of the human body, is known to provide much useful information, such as health status [1,2] and human identity information [3]. Skin appearance can be viewed as a kind of texture surface, and skin texture analysis can be used in various applications. For example, in [2], skin texture analysis is applied to computer-aided diagnosis in dermatology, where the dermatologist can use the computational texture representation to make an initial diagnosis for the patient. Meanwhile, biomedical evaluation based on skin texture can provide some tests for topical skin treatments, which can be used to judge whether these treatments are effective or not in the early stages. In addition, skin texture analysis can be used to estimate human skin age [4,5].

With the rapid development of computer techniques, researchers have investigated the use of various biometric traits, including fingerprints [6–8], face [9,10], iris [11,12], retina [13,14], palmprints [15–18] and finger-knuckle-prints [19], *etc.*, for the purposes of personal authentication. Moreover, face [20,21] and gait [22] have been used for gender classification. In [21], the authors demonstrated that the SVM classifier is able to learn and classify gender from a set of hairless low resolution face images with high classification accuracy. For gait-based gender recognition, a number of combinations of gait components [22] are extracted to classify gender with the SVM classifier. Skin texture, as a potential biometric identifier to assist existing biometric traits, has also received certain attention in the past years. Based on the locally consistent property of the fingerprint skin tissue, Rowe [23] extracted texture features of the fingerprint skin for human identification while reducing the size of the fingerprint sensing area. Cula and Dana *et al.* [3,24,25] used the bidirectional texture function, which is analogous to the bidirectional reflectance distribution function, to model skin texture to assist face recognition. For each skin texture surface, the bidirectional texture function is sampled in multiple camera views and illumination directions. However, obtaining accurate bidirectional image measurements of skin texture surface is hard, because the skin surface is non-planar, non-rigid and can be stretched.

It can be observed that the human hand back skin has a clear and consistent texture pattern which is uniformly distributed over a large portion of hand back. Based on our daily life experience, we know that the hand based skin texture (HBST) pattern is not permanent and it will change over time. For example, young people will have finer (*i.e.*, smoother and smaller size of micro-cells) HBST than old people, while females will have much finer HBST than male. Nonetheless, over a relatively long period, the HBST of a person is stable. Based on [26], the changes in skin associated with age can be visualized by gloss and wrinkles, and thus some measurements of wrinkles, gloss and density of microgrooves of skin can be used for age estimation. In [26], the number of pixels in the binary image of the epidermal cross-section is used to estimate the age. From the curve of measured peripheral length *vs.* age in [26], one can see that the peripheral length changes little in 1–2 years, which means that skin texture can remain stable for a relatively long period. These motivated us to investigate the possibility that the HBST pattern can be used to aid personal identification and gender classification. Many biometric identifiers such as fingerprints, faces, iris and palmprints, *etc.*, have been proposed for human identification, and our goal is not to compete with those biometric identifiers, but to validate whether HBST has a sufficient level of accuracy so that it can be helpful to assist the existing biometric authentication techniques. Moreover, apart from biometric applications, as a specific kind of texture patterns, the established HBST dataset can also be used to evaluate the texture feature extraction and classification algorithms in the community of computer vision and pattern recognition.

In this paper, we study the use of HBST for personal identification and gender classification. To this end, an HBST imaging device was designed to capture HBST images. Since HBST is a type of fine scale feature, a high resolution (about 450 dpi) is set to capture the detailed texture patterns in hand back images. Different from the method in [3], where skin texture is modeled as a 3D texture and the bidirectional texture function is used to describe the skin appearance, we model HBST as a kind of 2D appearance texture because the hand back can be approximately viewed as a 2D plane. Therefore, we directly capture the HBST image using a CCD camera with the fixed position under the fixed illumination direction. Such a design makes the HBST image acquisition very efficient and feasible for the purpose of personal identification and gender classification. In the 3D model [24,25], multiple cameras and multi-illuminations are needed to collect samples, which makes the imaging system very complex. Compared with the 3D model, modeling the hand back skin surface with the 2D model makes our imaging system much easier to design and more convenient to collect samples. In addition, our goal is to analyze the texture pattern in hand back skin so that 2D modeling is more suitable.

By using the designed HBST imaging device (please refer to Section 2 for more details), an HBST image database is established, which consists of 1,920 images from 80 volunteers (160 hands). A texton learning based method is then proposed for HBST pattern classification. The HBST images are passed through a bank of filters, and a set of textons are learned from the filter responses with the sparse representation (SR) technique. Then, under the SR framework, the representation coefficient histograms of HBST images are computed and used for classification. The performance of the proposed method is evaluated by using the established HBST database in comparison with state-of-the-art texture classification schemes, including the multi-fractal spectrum [27], original LBP [28], dominant LBP [29], completed LBP [30] and *k*-means based texton learning method [31,32]. Experimental results demonstrated that HBST could achieve interesting personal identification and gender classification accuracy, which implies that HBST can be used to aid existing biometric authentication techniques and improve the overall performance.

In summary, the major contributions of this work lie in that we developed the HBST imaging device, established an HBST dataset and proposed a sparse texton learning based HBST texture classification method. We validated that HBST pattern has potential to do personal identification and especially gender classification. In addition, as a special type of texture images, the established HBST dataset is very challenging, and it provides a good platform to evaluate and develop high performance texture feature extraction and classification algorithms.

The rest of this paper is organized as follows: Section 2 introduces the design and structure of the HBST imaging device. Section 3 presents in detail the process of texton learning and feature extraction for HBST classification. In Section 4, the proposed method is validated on the established HBST database for personal identification and gender classification. Section 5 concludes the paper.

### 2. Hand Back Skin Texture Imaging System

**Figure 1.**
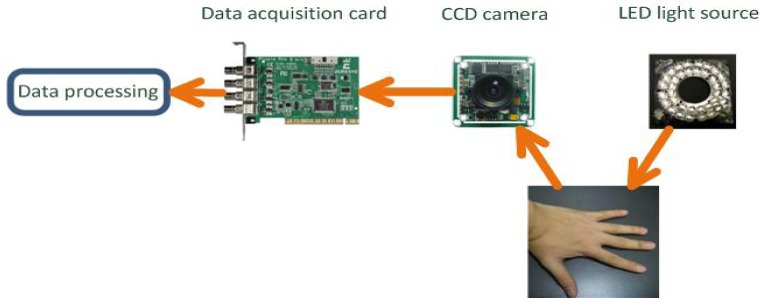
The schematic diagram of the developed hand back skin texture imaging system.

schematic diagram of the major components of the developed hand back skin texture (HBST) imaging system is shown in Figure 1. It is composed of a ring of LED light source, a lens, an associated CCD camera, and a data acquisition card. When it works to collect data, the LED light source will illuminate the hand back skin, and then the CCD camera will capture the HBST image and pass it to the data acquisition card. The data acquisition card will then transmit the image to the data processing unit (e.g., the CPU in a PC).

**Figure 2.**
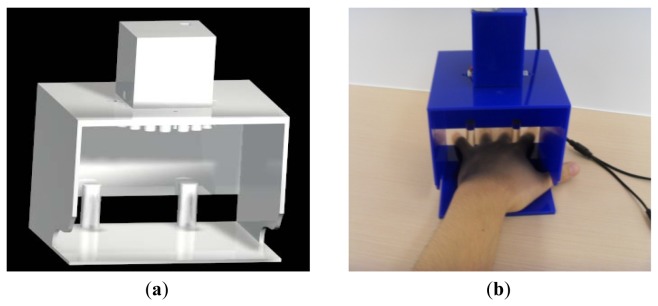
(**a**) The inner structure of the developed hand back skin texture imaging system; (**b**) The outside view of the imaging system.

Figure 2(a) illustrates the inner structure of the HBST imaging device and Figure 2(b) shows its exterior. A critical issue in HBST data acquisition is how to make the data collection environment as stable and consistent as possible so that undesired disturbances (e.g., the background and environmental illumination disturbances) can be reduced. Meanwhile, a stable data collection environment can effectively reduce the complexity of feature extraction and improve the classification accuracy. Specifically speaking, in our imaging system how to keep the illumination uniform and constant and how to fix the position of the hand are of the most importance. To this end, a ring of LED light source (in visible spectrum, 390–780 nm) and a CCD camera are enclosed in a box to keep the illumination nearly constant. The LEDs are arranged in a circle around the camera to make the illumination uniform. Referring to Figure 2(a), in order to capture the central part image of the hand back skin texture, two pegs are used to fix the hand, which can guide the position of index and little fingers with a user friendly interface. This can also reduce largely the pose variations of the hand in different capturing sessions. In addition, our design could make the skin texture surface as flat as possible so that we can model the skin surface as a 2D planar texture image. Note that there are some differences between our device and the palmprint device [16]. First, in order to capture the micro-structures of HBST, the resolution of the chosen camera in our device is higher than that in the palmprint device. Second, the light source is different from that in the palmprint device. In our device, the ring LED is used while the halogen light source is used in the palmprint device. Finally, the architecture of the device is different. In our HBST imaging system, we employ the micro-industrial CCD camera board, LED light source and USB data acquisition card to collect data. However, in the palmprint device, the commonly used industrial CCD camera, halogen light source and PCI data acquisition card are used to collect data. Compared to the palmprint device, the size of the HBST imaging device is much smaller due to the use of micro-industrial CCD camera board and LED light source. In addition, the cost of HBST device is also lower.

The texture pattern of human hand back skin can only be clearly observed in a relatively fine scale. In order to capture the HBST image in a high enough resolution while avoiding the HBST image size to be too big, the focal of the lens should be carefully designed. In our imaging system, due to the limited distance between the camera and the hand back, we chose to use a 12 mm focal length lens to capture the HBST images. Further reduction in the focal length will distort the captured image. The size of the CCD output image is 576 × 768 (the raw image is saved in the 24-bitmap format and we convert it into 8-bit gray level image), and finally the HBST image is captured under a resolution of about 450 dpi. In designing our imaging device, we tested different resolution settings of the HBST image, and found the resolution of about 450 dpi can satisfy our requirements. If the resolution of the image is too low, the micro-structures such as wrinkles in the image cannot be captured clearly. If the resolution of the image is too high, the cost of the camera will be high and the computational cost will also increase. A resolution of 450 dpi is good enough to capture clear HBST images at a low cost.

**Figure 3.**
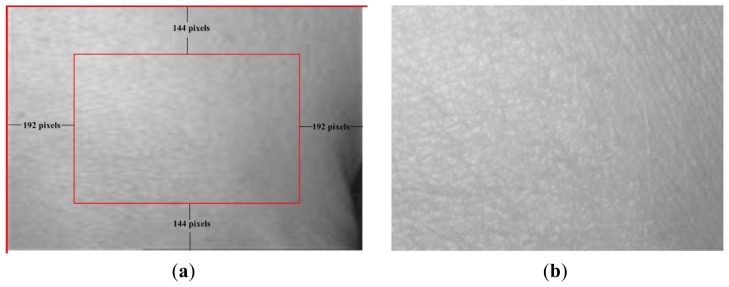
(**a**) is the raw image (size 576 × 768) captured by our device and (**b**) is the sub-image (size 288 × 384) cropped from the central part of (a).

In our HBST imaging system, since we use two pegs to fix the hand position, the top and bottom boundary of the captured skin texture image can be roughly fixed. Although the hand back skin can be viewed nearly as a 2D plane in the central part, the boundary part of the hand back can be quite distorted in the captured HBST image. In order to reduce the effect of the hand back boundary area on the later feature extraction and recognition procedures, we crop a sub-image from the captured raw image by removing the four boundary areas. Referring to Figure 3, we simply set the top left corner of the HBST image as the origin point, and based on our experimental experience we crop the central part of size 288 × 384 from the original image of size 576 × 768. Such a sub-image cropping process can not only make the feature extraction more stable and accurate, but also reduce computational cost.

**Figure 4.**
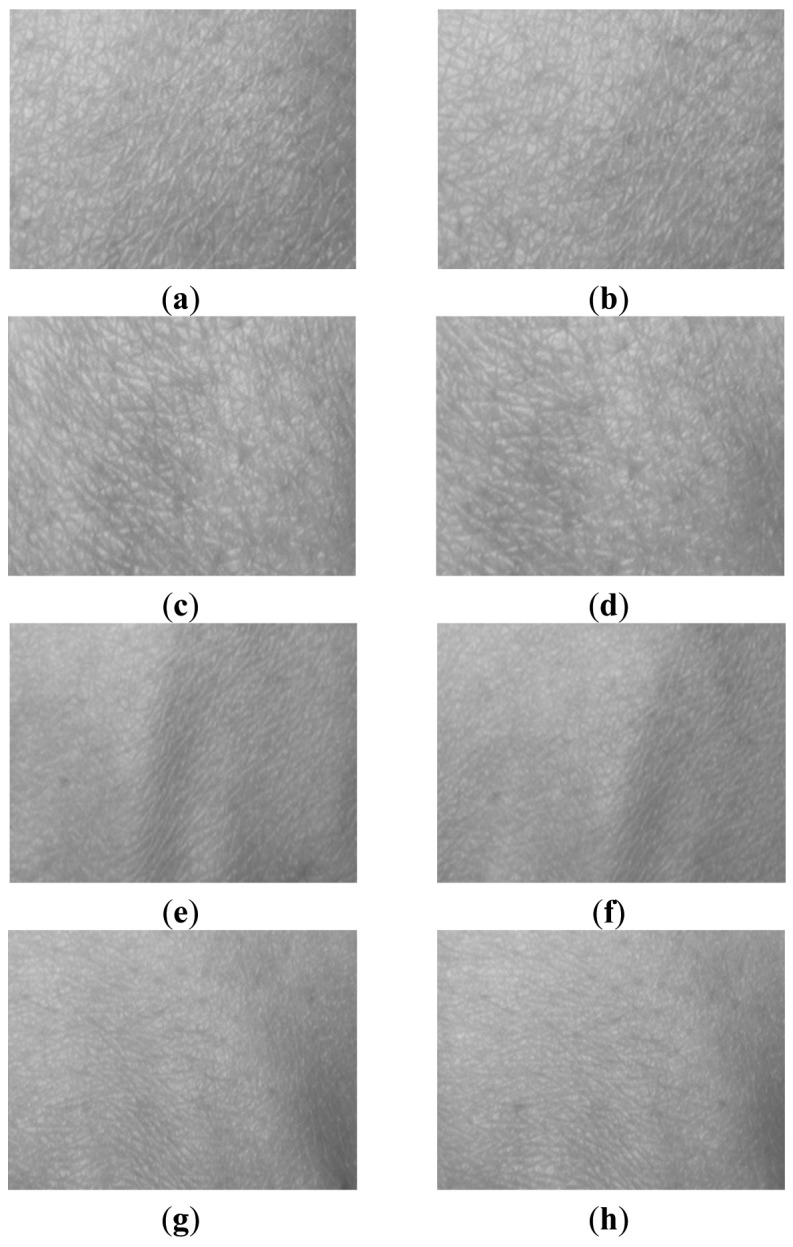
(**a**) and (**b**) are the cropped left-hand HBST images of a person collected in two different sessions, while (**c**) and (**d**) are the right-hand HBST images from the same person. (**e**) and (**f**) are the cropped left-hand HBST images from another person, while (**g**) and (**h**) are the right-hand HBST images from this person.

**Figure 5.**
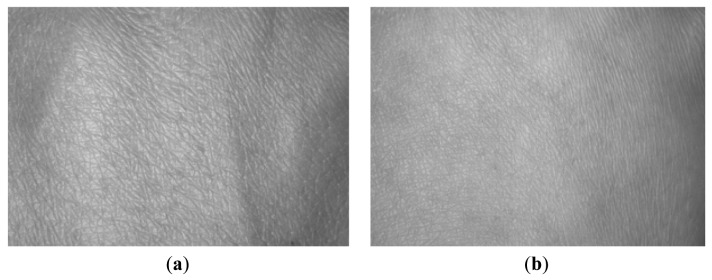
(**a**) and (**b**) are the HBST images from one male and one female, respectively.

Figure 4 shows some example cropped HBST images captured in two different sessions with a time interval of about 30 days. Figure 4(a,b) are the left-hand HBST images from one person in the two sessions, while Figure 4(c,d) are the right-hand HBST images from the same person. Figure 4(e,h) are the left and right-hand HBST images from another person. Figure 5 shows the HBST images from one male subject and one female subject. From these HBST example images, we can have the following observations. (1) First, the left-hand and right-hand HBST patterns of a person are similar. (2) Second, the HBST patterns captured in different sessions from the same person are similar. (3) Third, the HBST patterns from different persons are different, which implies its potential for human identification. (4) At last, the HBST patterns of male and female subjects are different, which makes HBST pattern a good feature for gender classification.

## 3. HBST Feature Extraction and Classification

Texture classification is a classical topic in computer vision and pattern recognition. Although some well-known texture classification methods [27,28,33] can obtain good performance on some benchmark databases such as the UIUC [33] and CUReT [34] texture databases, they may not be suitable for HBST patterns due to the special micro-structure of the hand back skin. The multi-fractal spectrum method [27] and the LBP method in [28] cannot obtain good results because the features generated by them cannot characterize the appearance of the skin texture well. Since there are no obvious interest points or interest regions in HBST images, the method in [33] cannot detect accurately the affine invariant regions for a robust statistical description of the skin texture, and thus it will fail to classify the HBST patterns.

With a more careful look of the HBST images, we can observe some properties of the HBST patterns. First, there are no clear edges and corner points in the HBST images. Second, the HBST patterns are made up of some micro-cellular structures. Third, those micro-structures are generally distributed uniformly across the whole HBST image. Based on these observations, we choose to learn the micro-structures (*i.e.*, textons) from the training HBST images, and then use them to describe the query HBST image for classification. Our experimental results in Section 4 also verify that the texton learning based method performs well for HBST pattern recognition.

As in [31,32], the texton learning is performed in the space of MR8 filter bank responses. Different from [31,32], which use the *k*-means method to learn the textons, in this paper we employ the technique of sparse representation (SR) [35,36] to learn an over-complete dictionary of textons via the *l*_1_-norm minimization. And under the SR framework, we extract the SR coefficient histogram as the HBST feature for recognition. By filtering the training images with the MR8 filter bank, for each class of HBST images we can construct a training dataset *X* = [*x*_1_, *x*_2_, …, *x_n_*], where *x_i_, i* = 1,2, …, *n*, is an 8-dimensional MR8 filtering response vector at a pixel of the training sample images of this class. A dictionary of textons, denoted by *D* = [*d*_1_, *d*_2_, …, *d_l_*], will be trained from the constructed training dataset *X*, where *d_j_, j* = 1,2, …, *l*, is a texton. The number of textons is generally much smaller than that of the elements in the training dataset, *i.e., l* ≪ *n*. In the following sub-sections, we present in detail the method for HBST feature extraction and classification.

### 3.1. MR8 Filter Bank

**Figure 6.**
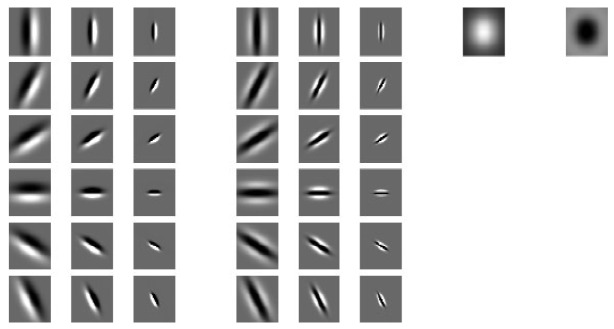
The MR8 filter bank.

The MR8 filter bank [31,32] is a nonlinear filter bank with 38 filters but only eight filter responses. It contains 36 bar and edge filters, which are along six orientations and across three scales, as well as a Gaussian filter and a Laplacian of Gaussian filter on a single scale. In order to obtain rotation invariance, for the edge and bar filters, the maximum filtering response along 6 orientations is selected for each scale. Moreover, using only the maximum orientation response can reduce the number of responses from 38 to 8. Figure 6 illustrates the MR8 filter bank. The motivation for using the MR8 filter bank is to extract rotation invariant skin texture features since there will be some rotation variations in the collected HBST images from the same subject. The MR8 filter bank responses are rotation invariant while preserving the distinctive features of the texture images.

When computing the MR8 filter bank responses, there are some pre-processing steps to follow in order to reduce some effects on feature extraction. Before convolving the original HBST image with the MR8 filter bank, all HBST images are normalized to have zero mean and unit standard deviation. This normalization can reduce the variations caused by illumination changes. After computing the MR8 filter responses, the filter response *x_i_* at pixel *i* is normalized using the Weber's law [31]: *x_i_*log(1+*L*/0.03)/*L*, where *L* = ‖*x_i_*‖_2_ is the magnitude of the filter response vector *x_i_*.

### 3.2. Texton Learning Based on SR

SR reveals the fact that if the input signal is intrinsically sparse in some domain, it can be sparsely represented over the dictionary which can define the sparse domain. For a given signal *x* ∈ *R^m^*, we say that *x* has a sparse approximation over a dictionary *D* = [*d*_1_, *d*_2_, …, *d_l_*], ∈ *R^m^*^×^*^l^*, if we can find a linear combination of only “a few” atoms from *D* that is “close” to the signal *x*. Under this assumption, the sparsest representation of *x* over *D* is the solution of the following minimization problem:

(1) $$\underset{\alpha }{\mathrm{argmin}}{\left\|\alpha \right\|}_{0}\;\text{s.t.}\;{\left\|x-D\alpha \right\|}_{2}^{2}\leq \varepsilon$$

where *α* ∈ *R^l^* is the sparse representation coefficient vector by coding signal *x* over dictionary *D* such that *x* ≈ *Dα* and most of the elements in *α* are close to zero. The *l*_0_-norm counts the number of non-zero elements in the represnetation vector *α*. Because the *l*_0_ -norm minimization is an NP hard problem, an alternative way is to solve the *l*_1_ -norm minimization problem:

(2) $$\underset{\alpha }{\mathrm{argmin}}{\left\|\alpha \right\|}_{1}\;\text{s.t.}\;{\left\|x-D\alpha \right\|}_{2}^{2}\leq \varepsilon$$

In the application of HBST analysis, the signal *x* is an 8-dimensional feature vector of MR8 filtering response at a pixel of the HBST image. In order to better represent the query image for the classification porpose, the dictionary *D* needs to be learned from the training HBST images. For each class of HBST images, we filter the training images with the MR8 filter bank and construct a training dataset *X* = [*x*_1_, *x*_2_, …, *x_n_*], where *x_i_, i* = 1,2, …, *n*, is the MR8 filtering response vector at pixel *i* of the training images of this class. The dictionary *D* assocaited with this class can be learned by optimizing the following objective function:

(3) $$\underset{D,\Lambda }{\mathrm{argmin}}{\left\|\Lambda \right\|}_{1}\;\text{s.t.}\;{\left\|X-D\Lambda \right\|}_{F}^{2}\leq \varepsilon$$

where *Λ* = [*α*_1_, *α*_2_, …, *α_n_*] and ‖‖*_F_* is the Frobenius matrix norm. We can rewrite Equation (3) into an unconstrainted optimization problem with a penalty term:

(4) $$\underset{D,\Lambda }{\mathrm{argmin}}{\left\|X-D\Lambda \right\|}_{F}^{2}+\lambda {\left\|\Lambda \right\|}_{1}$$

The optimization problem in Equation (4) is non-convex. Usually we can have a local minimum by alternatively optimizing *D* and *Λ*; that is, from some initialization of *D*, we can solve *Λ*, and then by fixing *Λ*, we can update *D*. Such a procedure iterates until convergence. In this paper, we adopt the alternating direction method in [37] to solve *Λ* (when *D* is fixed) and the Lagrange dual method [38] to update *D* (when *Λ* is fixed). After we learn the dictionary of textons for each class of HBST images, we combine these textons into one big over-complete dictionary, and use it to extract skin texture features.

In [31,32], the classical *k*-means clustering method is used to learn textons for texture image feature extraction and classification. Specificaly, the textons are determined by solving the following problem:

(5) $$\underset{d_{j}}{\mathrm{argmin}}\sum_{j=1}^{l}\sum_{x_{i}\in \Omega_{j}}{\left\|x_{i}-d_{j}\right\|}_{2}^{2}$$

The *k*-means clustering will partition the training set *X* = [*x*_1_, *x*_2_, …, *x_n_*] into *l* groups *Ω*_1_, *Ω*_2_, …, *Ω_l_*, and the texton *d_j_* is defined as the mean vector of all the vectors within *Ω_j_*. The *k*-means clustering based texton learning method can be viewed as a special case of the SR based dictionary learning. If we require that *α_i_* has only one non-zero element, which is 1, then the problem in Equation (5) will be basically the same as the problem in Equation (1). In this case, we use only one texton to represent the feature vector *x_i_* and assign the label of *x_i_* to that texton. For an input vector *x_i_* which may lie in the boundary of two or more clusters, the *k*-means clustering will randomly assign it to one of the classes. However, such a representation may not be accurate enough in practice. In contrast, by using SR, *x_i_* will be coded as a linear combination of more than one texton, which can achieve a much lower reconstruction error due to the less restrictive constraint. In the experiments in Section 4, we will see that by using the SR technique to learn the textons and the associated feature description method in Section 3.3, the HBST recognition accuracy can be much improved.

### 3.3. Feature Extraction and Classification Based on Learned Textons

Denote by D*_k_* the texton dictionary for the *k*th class of HBST, the dictionary for all *c* classes of HBST images can be formed by amalgamating the *c* dictionaries, *D* = [*D*_1_, *D*_2_, …, *D_c_*]. With this dictionary *D*, each training HBST image can generate a model by mapping it to the texton dictionary.

In the method of [31,32], each pixel of a texture image, is labeled with the element in the dictionary *D* that is closest to the feature vector at this pixel. A histogram of texton labels of this image is then formed for classification. Different from this method, under the SR framework, we can construct a histogram of the SR coefficients of a texture image for classification. The representation coefficient vector can be obtained by coding the feature vector *x_i_* over *D* with the SR technique. However, the computational cost of solving the *l*_1_-norm minimization problem to obtain the SR coefficient is very heavy because of the highly over-complete dictionary *D*. To reduce the cost of sparse coding, we can use only a subset of *D* to represent *x_i_*. Specifically, as in [39] we use the closest *t* textons (*t* ≪ *z, z* is the total number of textons learned from the *c* classes of HBST images) to *x_i_* in *D* to form a sub-dictionary for *x_i_*. Denote by $d_{1}^{i},d_{2}^{i},\ldots ,{\text{d}}_{t}^{i}$ the *t* closest textons to *x_i_*, and the sub-dictionary for *x_i_* is then $D_{i}=[d_{1}^{i},d_{2}^{i},\ldots ,{\text{d}}_{t}^{i}]$. The representation vector of x*_i_* over *D_i_*, denoted by $\alpha_{i}=[\alpha_{1}^{i},\alpha_{2}^{i},\ldots ,\alpha_{t}^{i}],$ can then be computed by solving the following *l*_1_-norm minimization problem:

(6) $$\underset{\alpha_{i}}{\mathrm{argmin}}{\left\|x_{i}-D_{i}\alpha_{i}\right\|}_{F}^{2}+\lambda {\left\|\alpha_{i}\right\|}_{1}$$

The alternating direction method in [37] can be used to solve Equation (6). Since *D_i_* is a subset of *D*, once we have, *α_i_* we can easily construct another representation vector *h_i_* over *D* such that:

(7) $$D_{i}\alpha_{i}=Dh_{i}$$

Obviously, most of the entries in *h_i_* will be 0, and only the entries corresponding to the same textons as those in *D_i_* will have non-zeros values, which are the same as those in *α_i_*.

**Figure 7.**
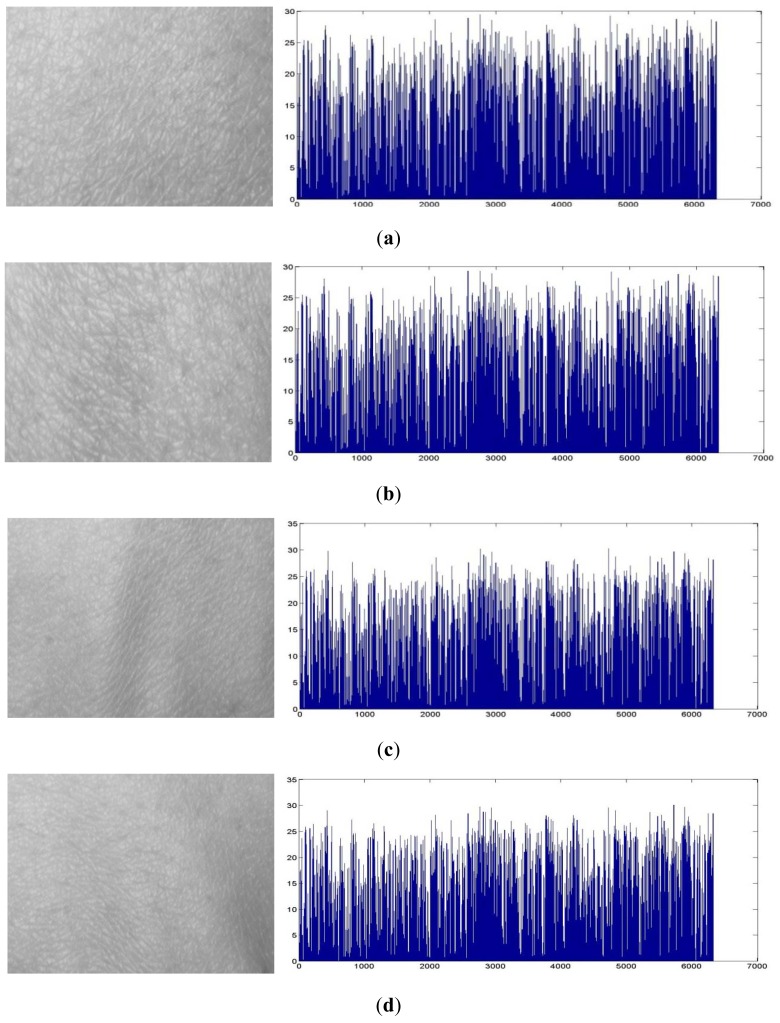
The coefficient histograms of HBST images from different persons. (**a**) and (**b**) are the histograms of the left-hand HBST images from the same person while (**c**) and (**d**) are the histograms of the left-hand HBST images from another person.

Finally, for each pixel at position *i*, we have a representation vector. *h_i_*. Hence, we can form a representation coefficient histogram, denoted by *H_f_*, for this HBST image by summing all the vectors of |*h_i_*|:

(8) $$H_{f}=\sum_{i=1}^{N}\left|h_{i}\right|$$

where *N* is the number of pixels in the HBST image. The *H_f_* can be taken as the final feature descriptor of the HBST image for the classification purpose. Figure 7 shows the coefficient histograms of some HBST images from different persons.

We denote by *H_j_, j* = 1, 2, …, *J*, the histogram of a training texture image. Similarly, for an input test image *Y*, we can construct a representation coefficient histogram for it, denoted by *H_Y_*. The similarity between *H_j_* and *H_Y_* can be computed as:

(9) $$\chi^{2}(H_{j},H_{Y})=\frac{1}{2}\sum \frac{{\left(H_{j}-H_{Y}\right)}^{2}}{H_{j}+H_{Y}}$$

The test HBST image *Y* can then be classified with the nearest neighbor classifier. That is, it is classified to the class whose training sample has the shortest *χ*^2^ distance to it.

## 4. Experimental Results

### 4.1. Database Establishment

In order to evaluate the proposed HBST analysis method for personal identification and gender classification, we established an HBST image database using the developed HBST imaging device. Those HBST sample images were collected from 80 volunteers (160 hands), including 61 males and 19 females whose ages ranged from 20 to 50 years old.

The samples were collected in two different sessions. In each session, each person was asked to provide six left-hand and six right-hand HBST images, respectively. Therefore, 12 samples from one person were collected in each session. In total, the database contains 1,920 samples from 160 hand backs. The average interval between the first and second session is about 30 days, and the maximum and minimum intervals are 40 days and 14 days, respectively. In the following experiments, without specific instructions, we use the samples collected in the first session as the training set and the samples in the second session as the test set.

Due to the various difficulties in data collection (e.g., the funding support, the recruitment of volunteers, *etc.*), our established database may not be large and comprehensive enough to support very strong conclusions. Nonetheless, we believe that its size is reasonably large to illustrate if HBST patterns can be used to assist personal identification and gender classification. We are planning to collect more samples from more subjects in the following years, making our database more comprehensive and more balanced in terms of male and female subjects.

### 4.2. Personal Identification

In this section we aim to answer the question that whether HBST can be used as a kind of biometric trait to aid personal identification. To this end, we conducted five experiments using the proposed texton learning method with SR (TL_SR), and we compare the proposed TL_SR method to some representative texture classification methods such as the multi-fractal spectrum method [27], original LBP [28], dominant LBP (DLBP) [29], completed LBP (CLBP) [30] and the texton learning method using the *k*-means clustering (TL_KM) [31]. For the multi-fractal spectrum method, the dimension of the multi-fractal spectrum vector is set as 26. In the original LBP, dominant LBP and completed LBP method, the radius of the neighborhood is set to 2 and the number of sampled points in the neighborhood is set to 8. For TL_KM, 40 textons are learned for each class of HBST images. In the proposed TL_SR method, 40 textons are also learned per class. Moreover, in the stage of feature description, for each descriptor *x_i_, t* is set as 100, which means that 100 closest textons to *x_i_* in *D* are chosen to form a sub-dictionary to obtain the SR coefficient. In the following experiments, we use the classification accuracy to evaluate these HBST classification methods. The classification accuracy is computed as *r* = *n_c_*/*n*, where *n_c_* is the number of correctly classified test samples and *n* is the number of all test samples.

#### 4.2.1. Experiment 1

In the first experiment, all classes of HBST images are involved. The left and right hand HBST images from the same person are taken as from different classes. Therefore, in this experiment, there are 160 classes and each class has six training and six test samples. Since the multi-fractal spectrum vector and the histogram generated by the original LBP method cannot characterize well the appearance (e.g., cell-like micro-structures) of skin texture, they lead to poor experimental results in our task. The multi-fractal spectrum and original LBP methods can only achieve the classification accuracy of 35.65% and 46.52%, respectively. Hence, in the following experiments, we only compare TL_SR with DLBP, CLBP and TL_KM.

Table 1 shows the classification accuracies by the competing methods. We can see that the TL_SR method that uses the SR coefficient histogram as feature is superior to the TL_KM method that uses the texton label histogram for HBST classification. Also, the proposed method is better than the CLBP method, which combines the central pixel, magnitude and sign information of the neighborhood to completely model the LBP operator.

The interesting HBST image classification accuracies validate that the proposed HBST identification system can well capture the characteristics of skin textures, allowing good discrimination between different classes. These results also suggest that human identification can be aided by HBST analysis.

#### 4.2.2. Experiment 2

In the second experiment, all HBST images are involved. Different from Experiment 1, here the left and right hand HBST images from the same person are viewed as from the same class. Therefore, in this experiment there are 80 classes and each class has 12 training and 12 test samples. The experimental results using the DLBP, CLBP, TL_KM and TL_SR method are compared in Table 2. We can see that for all methods the classification accuracy is increased. This is mainly because the total number of classes is smaller than that in Experiment 1, and the left hand and right hand HBST images of one person are similar.

#### 4.2.3. Experiment 3

The aim of this experiment is to evaluate the performance on the left and right hand HBST separately. For either left hand or right hand HBST images, there are 80 classes and 480 images in the training and test sets, respectively. The classification accuracies by different methods are listed in Tables 3 and 4. From the experimental results, one can see that the classification accuracy on the right-hand HBST images is slightly higher than that on the left-hand HBST. This is probably because most people who provided their HBST samples to our database are right handed so that they feel more convenient to use our imaging device with the right hand. Therefore, compared to the left-hand HBST samples, the right-hand HBST samples collected in our database have less deformation, which results in a slightly higher classification accuracy for personal identification.

#### 4.2.4. Experiment 4

In this experiment, we fuse the left-hand and right-hand HBST for identification. That is, both the left-hand and right-hand HBST samples of a person will be collected to identify his/her identity. Therefore, there are 480 pairs of left-hand and right-hand samples in the training set, which are from 80 subjects. In the test set there are also 480 pairs of HBST samples. For the left-hand and right-hand test samples, we calculate two distances $\chi_{l}^{2}$ and $\chi_{r}^{2}$, where $\chi_{l}^{2}$ is the distance between the left-hand test sample and left-hand training sample, and $\chi_{r}^{2}$ is the distance between the right-hand test sample and right-hand training sample from the same pair. Then the two distances can be fused by the simple weighted average method. The final distance for classification is $\chi_{f}^{2}=w\times \chi_{l}^{2}+(1-w)\times \chi_{r}^{2}$, where the weight *w* can be trained from the training dataset using the “leave-one-out” strategy. For the four competing classification methods in our experiment, the weights are 0.4, 0.5, 0.45 and 0.4, respectively. The classification accuracies by fusing the left-hand and right-hand HBST with different methods are listed in Table 5. Compared with the results in Experiments 1∼3 (please refer to Tables 1∼4), one can see that the classification accuracy by fusing the left-hand and right-hand HBST images is much increased, showing that the left-hand and right-hand HBST patterns have complementary information.

#### 4.2.5. Experiment 5

As we mentioned in the Introduction section, one goal of this work is to investigate whether hand back skin texture patterns can be used to aid other biometrics identifiers to improve personal identification accuracy. Therefore, in this experiment we fuse palmprint and HBST for personal identification. Since there are 160 hand backs (80 left hands and 80 right hands) in our HBST dataset, we randomly extract from the PolyU palmprint database [16] 1,920 palmprint images, which belong to 160 palms (80 left hands and 80 right hands). Each palm has 12 samples collected from two separated sessions, 6 samples per session. We then assume that each hand has six palmprint images and six HBST images in each session, and use the data from the first session for training, and use the data from the second session for testing.

We use the competitive code scheme [40] to extract the palmprint feature, and use the Hamming distance to measure the similarity between palmprint features. As in Section 4.2.4, we fuse the palmprint and HBST matching distances by the weighted average method. The final distance for classification is *d_f_* = *w* × *d_p_* + (1 − *w*) × *d_h_*, where *d_p_* is the distance between palmprint samples and *d_h_* is the distance between HBST samples. In our experiment, the weight *w* is set to 0.8 by experience. The classification accuracies of palmprint, HBST and the fusion of palmprint and HBST are listed in Table 6. Compared with the identification rate by either palmprint or HBST individually, one can see that the accuracy is improved by fusing palmprint and HBST matching distances. This validates that HBST can be used to aid the existing biometric traits for personal identification.

### 4.3. Gender Classification

As can be seen in Figure 5, the hand back skin appearance differs much from male to female. In most cases, the HBST surface from female is much smoother than that from male, and size of micro-cells in female HBST samples is smaller than those for males. Therefore, it is very interesting to verify that if the HBST patterns are distinctive enough to distinguish males from females. In this section, we conduct such experiments for gender classification.

In our HBST database, there are 61 males and 19 females. In gender classification, there are only two classes: male and female. The samples from all the 61 male subjects are taken as the samples of the male class, and the samples from all the 19 females are taken as those of the female class. The 960 samples collected from the first session are used as the training set, and the other 960 samples from the second session are taken as test samples. Table 7 shows the results by the DLBP, CLBP, TL_KM and TL_SR method. One can see that the gender classification accuracy can be higher than 98%, which implies that HBST can be aided to distinguish males from females.

Moreover, in Table 8 we present the numbers of falsely classified male and female samples by the proposed TL_SR method. As illustrated in Table 8, among the 732 male test samples, nine samples are incorrectly classified. Among the 228 female samples, four samples are falsely classified. The classification error rates of male and female samples are 1.23% and 1.75%, respectively. Although the numbers of male and female subjects in our database are not balanced, the classification error rate on female samples is only slightly higher than that on male samples. Certainly, we need to collect more samples and make the dataset more balanced to further validate this conclusion.

### 4.4. Discussion

Currently, compared with the biometric traits such as fingerprints, iris scans and palmprints, *etc.*, the personal identification accuracy of HBST is much lower than for those. However, each biometric trait has its pros and cons, and no one can supersede another one. In practice using two or more biometric traits together will provide a more robust solution. In this work, our goal is to investigate whether hand back skin texture patterns can be used to aid personal identification and gender classification. Considering that HBST images can be collected when capturing fingerprint or palmprint images, fusing fingerprint/palmprint and HBST can be a good way for multi-modal biometrics, as we demonstrated in Section 4.2.5.

Furthermore, as a specific type of texture images, the established HBST dataset can be used to test texture classification algorithms in the community of computer vision and pattern recognition. Different from the commonly used texture datasets such as UIUC [33], CUReT [34] and KTH_TIPS [41], which are challenging in terms of scaling, viewpoint and illumination variations, the established HBST dataset is also challenging but in a very different aspect: the high inter-class similarity. In CUReT, KTH_TIPS and UIUC, different materials are viewed as different classes. However, in our HBST dataset, samples are from different persons but they are all from the same material: skin texture. Although there are no significant scale, viewpoint and illumination changes in the HBST dataset, the high inter-class similarity makes it challenging to achieve a high classification rate. Some classical texture classification methods such as LBP and multi-fractal spectrum, which work well on the CUReT, KTH_TIPS and UIUC datasets, do not work well on the HBST dataset. This motivates us to develop more advanced texture classification methods.

It should be noted that although HBST analysis can assist personal identification and gender classification, there are some factors, such as hairs on skin and humidity of skin, to affect the performance of personal identification and gender classification. In our established HBST database, most of samples are collected from oriental people so that there are relatively few hairs on the hand back skin. In our future work, we will collect more samples from more subjects and investigate the influences of these factors on skin texture analysis. In addition, modeling skin texture over a long period is a challenging problem since there are large variations between skin textures in different ages. Hence, in the future we will study how to model skin texture over a long period more effectively to improve the performance of biometric tasks with skin texture analysis.

## 5. Conclusions

This paper studied the problem of using hand back skin texture (HBST) for assisting personal identification and gender classification. An effective skin texture imaging system was developed for capturing HBST images. Moreover, we employed the sparse representation (SR) technique to learn the dictionary of textons to model the HBST pattern. Then, based on the learned textons of HBST images, we extracted the SR coefficient histogram as feature for classification. To evaluate the performance of the proposed system, an HBST database was established, consisting of 1,920 images from 160 hands of 80 persons. Extensive experiments were conducted and the experimental results showed that human identification and gender classification can be aided by HBST analysis with good performance. In the future, more HBST samples need to be collected to verify the different aspects of HBST analysis and algorithm development. Meanwhile, some factors (hairs on the skin, humidity, *etc.*) will be investigated for HBST analysis.

## Figures and Tables

**Table 1.** Classification accuracies by competing methods. For one person, the left hand and right hand HBST images are viewed as from two different classes. Thus there are 160 classes in this experiment.

**Method**	**DLBP** [29]	**CLBP** [30]	**TL_KM** [31]	**TL_SR**

Accuracy	75.56%	84.51%	84.40%	86.81%

**Table 2.** Classification accuracies by competing methods. For one person, the left hand and right hand HBST images are viewed as from the same class. Thus there are 80 classes in this experiment.

**Method**	**DLBP** [29]	**CLBP** [30]	**TL_KM** [31]	**TL_SR**

Accuracy	78.59%	86.29%	88.40%	90.17%

**Table 3.** Classification accuracies on the left-hand HBST images.

**Method**	**DLBP** [29]	**CLBP** [30]	**TL_KM** [31]	**TL_SR**

Accuracy	80.38%	85.51%	84.54%	88.60%

**Table 4.** Classification accuracies on the right-hand HBST images.

**Method**	**DLBP** [29]	**CLBP** [30]	**TL_KM** [31]	**TL_SR**

Accuracy	82.91%	86.44%	85.24%	89.71%

**Table 5.** Classification accuracies by fusing the left hand and right hand HBST.

**Method**	**DLBP** [29]	**CLBP** [30]	**TL_KM** [31]	**TL_SR**

Accuracy	85.23%	87.24%	89.03%	92.51%

**Table 6.** Classification accuracies by palmprint, HBST and the fusion of them.

**Feature**	**Palmprint**	**HBST**	**Fusion**

Accuracy	98.65%	86.81%	99.58%

**Table 7.** Gender classification accuracies by different methods.

**Method**	**DLBP** [29]	**CLBP** [30]	**TL_KM** [31]	**TL_SR**

Accuracy	95.46%	97.63%	98.60%	98.65%

**Table 8.** Numbers and rates of falsely classified male and female samples by the proposed TL_SR method.

	**Male**	**Female**

Number	9	4
Rate	1.23%	1.75%

## References

[1] Skin disease atlas. http://www.dermnet.com.

[2] Cula O.G., Dana K.J., Murphy F.P., Rao B.K. (2004). Bidirectional imaging and modeling of skin texture. IEEE Trans. Biomed. Eng..

[3] Cula O.G., Dana K.J., Murphy F.P., Rao B.K. (2005). Skin texture modeling. Int. J. Comput. Vis..

[4] Tanaka H., Nakagami G., Sanada H. (2008). Quantitative evaluation of elderly skin based on digital image analysis. Skin Res. Technol..

[5] Kim K., Choi Y., Hwang E. Wrinkle Feature-Based Skin Age Estimation Scheme.

[6] Jain A.K., Flynn P., Ross A. (2007). Handbook of Biometrics.

[7] Maltoni D., Maio D., Jain A.K., Prabhakar S. (2009). Handbook of Fingerprint Recognition.

[8] Ratha N., Bolle R. (2004). Automatic Fingerprint Recognition Systems.

[9] Delac K., Grgic M. (2007). Face Recognition.

[10] Wechsler H. (2006). Reliable Face Recognition Methods-System Design, Implementation and Evaluation.

[11] Daugman J. (1993). High confidence visual recognition of persons by a test of statistical independence. IEEE Trans. Pattern Anal. Mach. Intell..

[12] Daugman J. (2004). How iris recognition works. IEEE Trans. Circuits Syst. Video Technol..

[13] Hill R.B. (1999). Retinal Identification, in Biometrics: Personal Identification in Networked Society.

[14] Borgen H., Bours P., Wolthusen S.D. Visible-Spectrum Biometric Retina Recognition.

[15] Guo Z., Zhang D., Zhang L., Zuo W.M. (2009). Palmprint verification using binary orientation co-occurrence vector. Pattern Recognit. Lett..

[16] Zhang D., Kong W.K., You J., Wong M. (2003). Online palmprint identification. IEEE Trans. Pattern Anal. Mach. Intell..

[17] Kong A., Zhang D., Kamel M. (2006). Palmprint identification using feature-level fusion. Pattern Recognit..

[18] Sun Z.N., Tan T.N., Wang Y.H., Li S.Z. Ordinal Palmprint Representation for Personal Identification.

[19] Zhang L., Zhang L., Zhang D., Zhu H.L. (2010). Online finger-knuckle-print verification for personal authentication. Pattern Recognit..

[20] Bruce V., Burton A.M., Dench N., Hanna E., Healey P., Mason O., Coombes A., Fright R., Linney A. (1993). Sex discrimination: How do we tell the difference between male and female faces?. Perception.

[21] Moghaddam B., Yang M.H. (2002). Learning gender with support faces. IEEE Trans. Pattern Anal. Mach. Intell..

[22] Li X., Maybank S.J., Yan S., Tao D., Xu D. (2008). Gait components and their application to gender classification. IEEE Trans. Syst. Man Cybern. Part C Appl. Rev..

[23] Rowe R.K. Biometrics Based on Multispectral Skin Texture.

[24] Cula O.G., Dana K.J. Compact Representation of Bidirectional Texture Functions.

[25] Cula O.G., Dana K.J. (2004). 3D texture recognition using bidirectional feature histograms. Int. J. Comput. Vis..

[26] Tatsumi S., Noda H., Sugiyama S. (1999). Estimation of age by epidermal image processing. Leg. Med..

[27] Xu Y., Ji H., Fermuller C. A Projective Invariant for Textures.

[28] Ojala T., Pietikainen M., Maenpaa T. (2004). Multi-resolution gray-scale and rotation invariant texture classification with local binary patterns. IEEE Trans. Pattern Anal. Mach. Intell..

[29] Liao S., Law M.W.K., Chung A.C.S. (2009). Dominant local binary patterns for texture classification. IEEE Trans. Image Process..

[30] Guo Z., Zhang L., Zhang D. (2010). A completed modeling of local binary pattern operator for texture classification. IEEE Trans. Image Process..

[31] Varma M., Zisserman A. Classifying Images of Materials: Achieving Viewpoint and Illumination Independence.

[32] Varma M., Zisserman A. (2005). A statistical approach to texture classification from single images. Int. J. Comput. Vis..

[33] Lazebnik S., Schmid C., Ponce J. (2005). A sparse texture representation using local affine regions. IEEE Trans. Pattern Anal. Mach. Intell..

[34] Dana K.J., Ginneken B., Nayar S.K., Koenderink J.J. (1999). Refelctance and texture of real world surfaces. ACM Trans. Graph..

[35] Donoho D. (2006). For most large underdetermined systems of linear equations the minimal l1-norm solution is also the sparsest solution. Commun. Pure Appl. Math..

[36] Candes E., Romberg J., Tao T. (2006). Stable signal recovery from incomplete and inaccurate measurements. Commun. Pure Appl. Math..

[37] Yang J., Zhang Y. (2011). Alternating direction algorithms for l1-problems in compressive sensing. SIAM Sci. Comput..

[38] Lee H., Battle A., Raina R., Ng A.Y. Efficient Sparse Coding Algorithms.

[39] Xie J., Zhang L., You J., Zhang D. Texture Classification via Patch-Based Sparse Texton Learning.

[40] Kong A., Zhang D. Competitive Coding Scheme for Palmprint Verification.

[41] Hayman E., Caputo B., Fritz M., Eklundh O. On the Significance of Real-World Conditions for Material Classification.

